# Supplementary material for: Anti-Virulence Properties of Plant Species: Correlation between In Vitro Activity and Efficacy in a Murine Model of Bacterial Infection
Source: Microorganisms. 2021 Nov 25;9(12):2424. doi: 10.3390/microorganisms9122424 (PMC8706108; doi:10.3390/microorganisms9122424)
Supplement: Supplementary file 1 [file microorganisms-09-02424-s001.zip › microorganisms-1426780-supplementary.pdf]

Supplementary Material

Anti-virulence properties of plant species: correlation between *in vitro* activity and efficacy in a murine model of bacterial infection

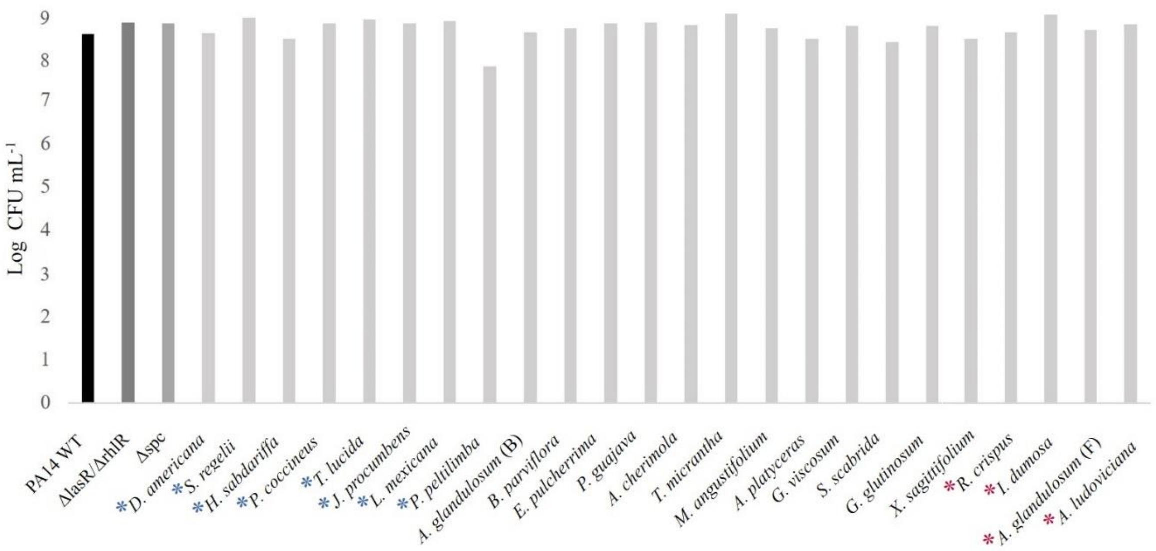

Figure S1. Effect of plant extracts on the viability of *P. aeruginosa* PA14 WT. Before inoculation in the animals, the bacteria were incubated with the extracts for 20 minutes at room temperature. The viability of the bacteria was determined by plate count. Blue dots indicate the extracts that reduced pathogenicity in the animal model and red dots those that stimulated it.

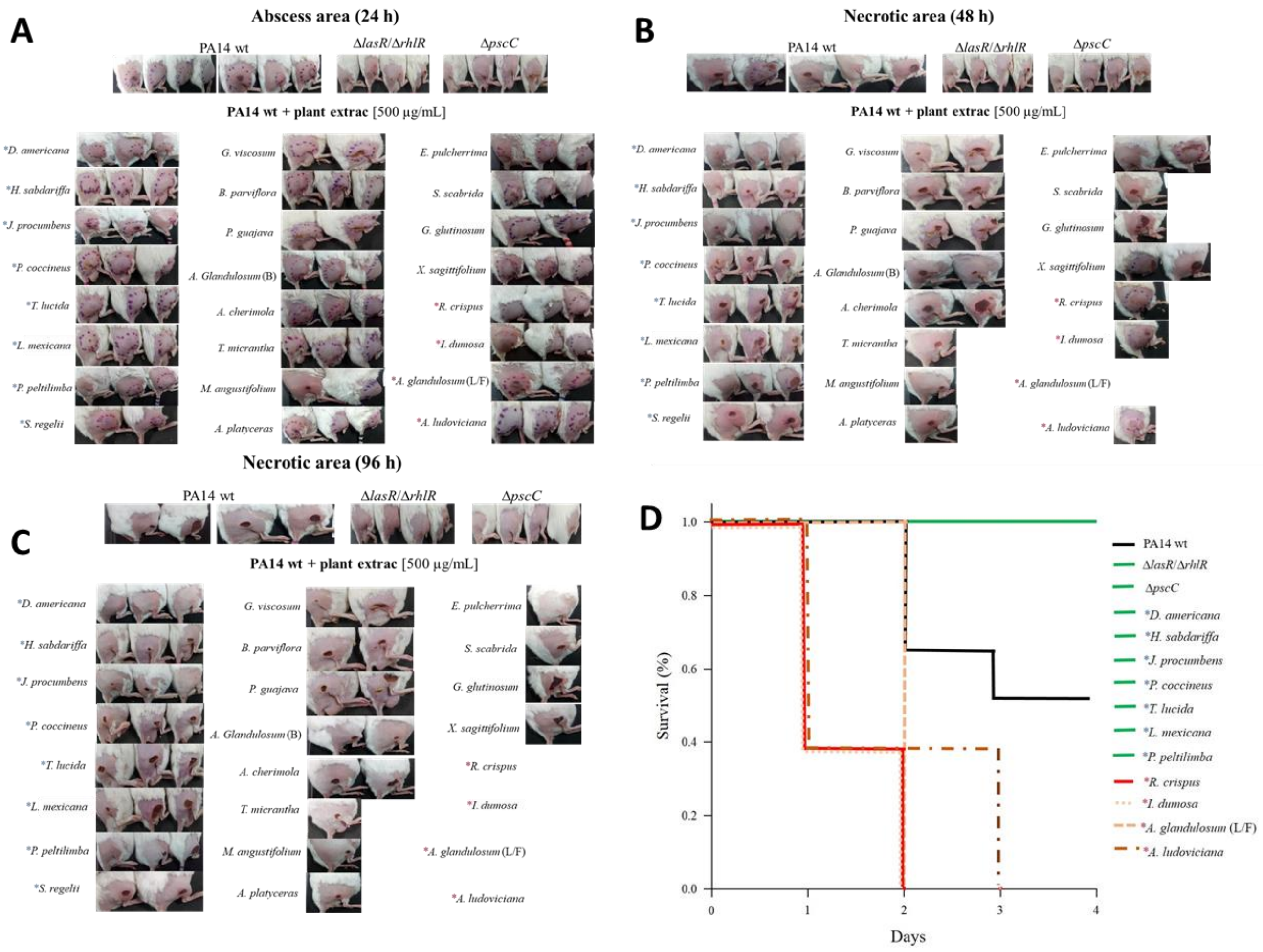

Figure S2. Images of the experiment in Table 2. Abscess formation at 24 h (**A**) and necrosis at 48 h (**B**). Survival was measured up to 96 h (**C**). Kaplan-Meier survival curve (**D**). The animals were sacrificed to quantify the presence of the bacteria in the tissues. The experiment was conducted once with groups of three animals per treatment, except the control with PA14 WT, which were two independent tests with three and four animals.

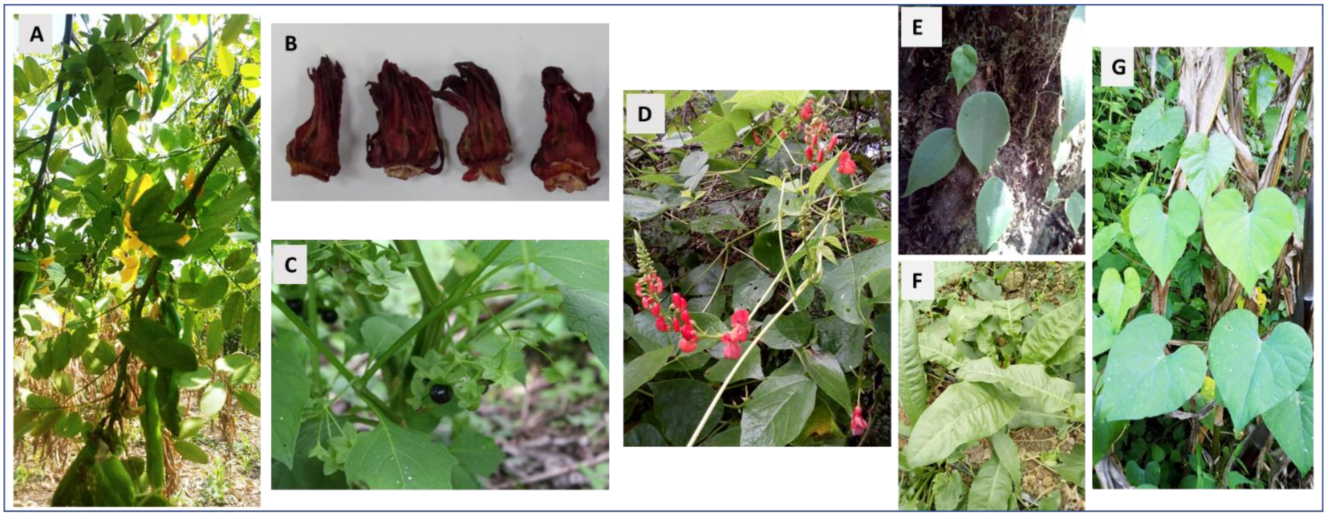

Figure S3. **A**: *D. americana*; **B**: *H. sabdariffa*; **C**: *J. procumbens*; **D**: *P. coccineus*; **E**: *P. peltilimba*; **F**: *R. crispus* and **G**: *I. dumosa*.

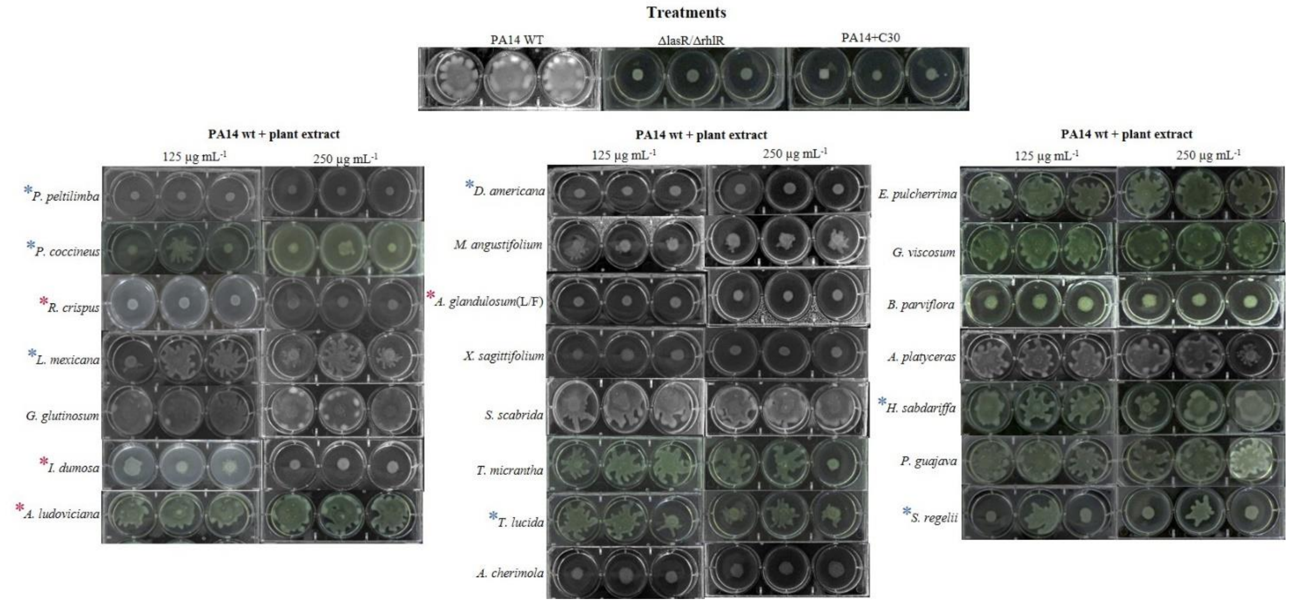

Figure S4. Representative images of the effect of plant extracts (125 and 250 μg/mL) on *P. aeruginosa* swarming. C-30, furanone C30 (50 μM).

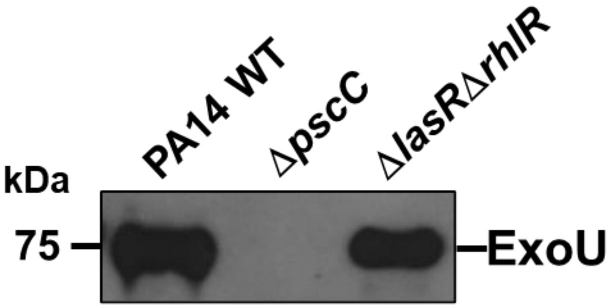

Figure S5. ExoU protein secretion (Western blot) in *P. aeruginosa* mutant strains. Representative image of three independent experiments.

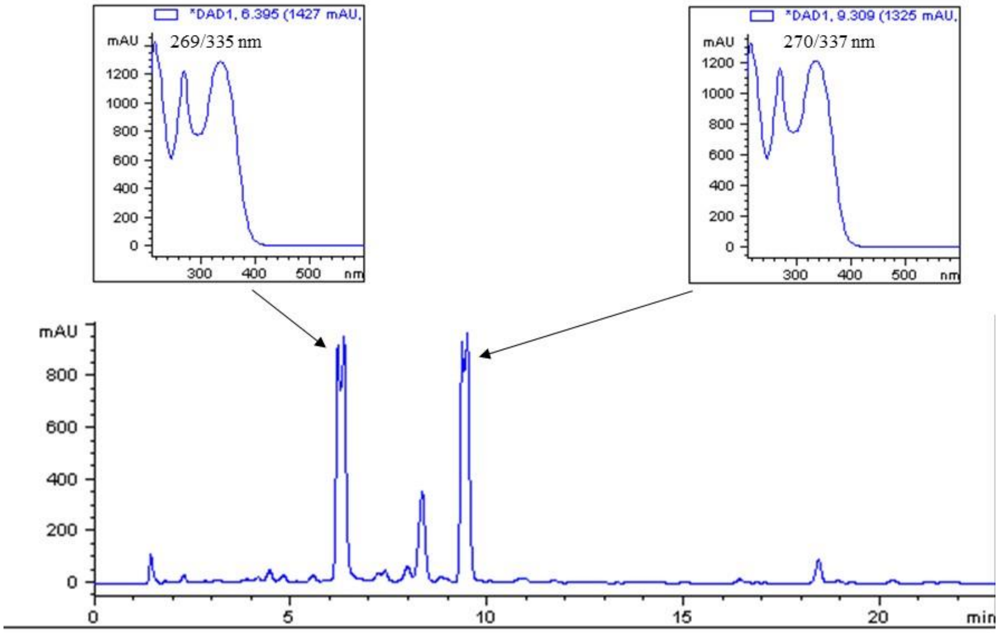

Figure S6. HPLC chromatogram showing two peaks corresponding to possible flavones from the dichloromethane extract of *D. americana*.

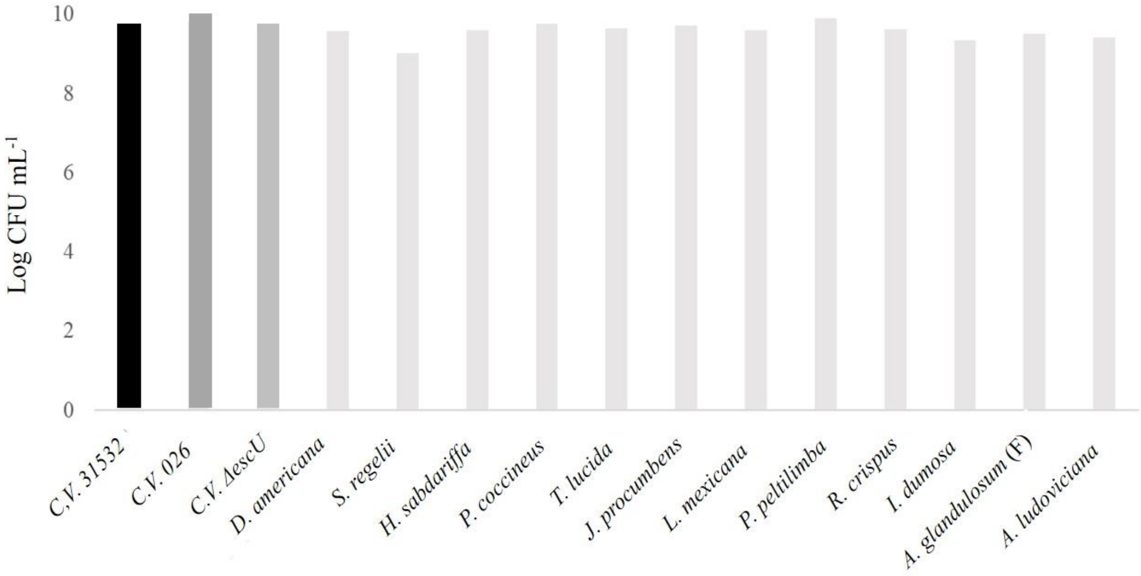

Figure S7. Effect of dichloromethane extracts on the viability of *C. violaceum* 31532 wt. Before inoculation in the animals, the bacteria were incubated with the extracts for 20 minutes at room temperature. Viability was determined through a standard plate count.

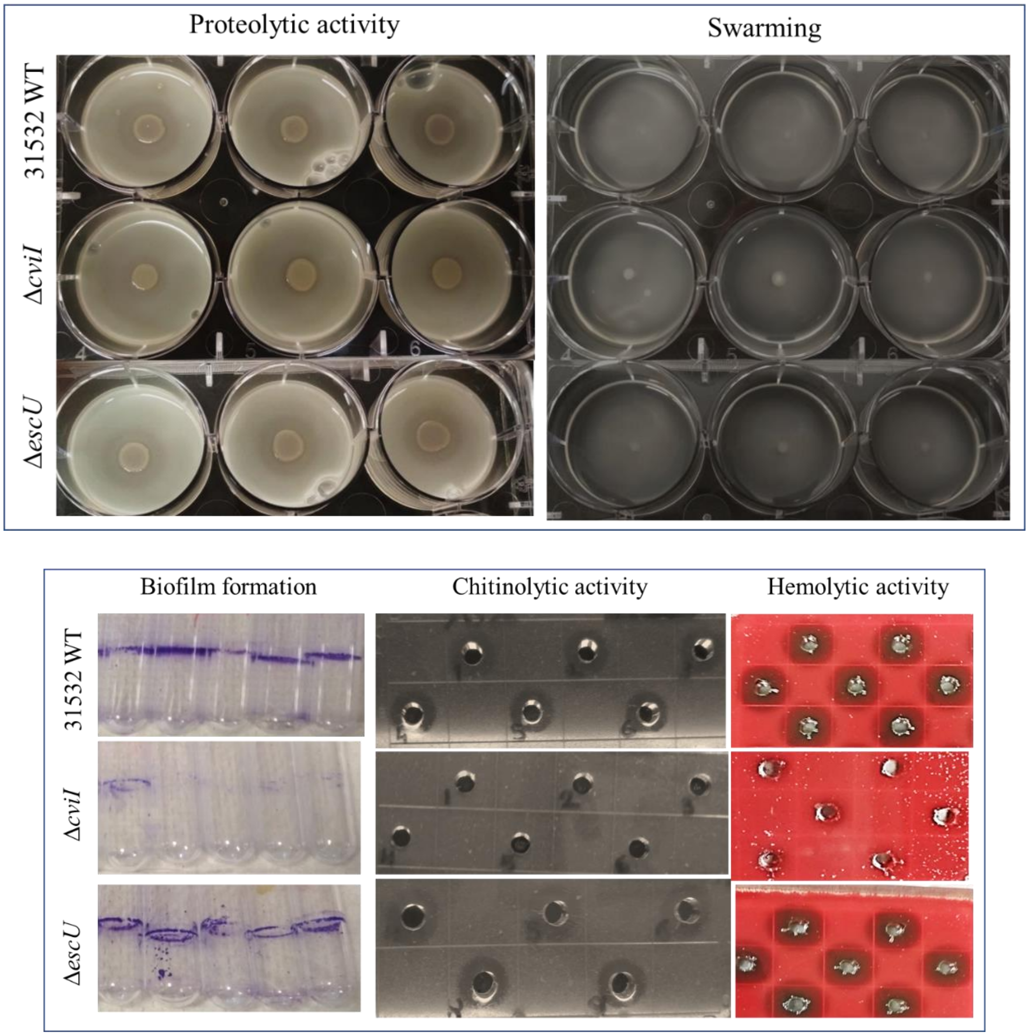

Figure S8. Effect on the production of virulence factors in QS and T3SS mutant strains of *C. violaceum.*

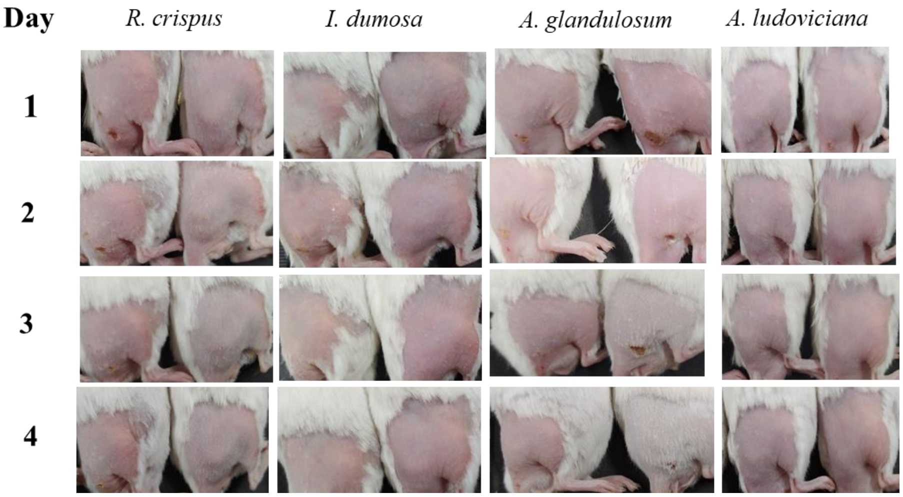

Figure S9. Images of the areas where the dichloromethane extracts without bacteria (PA14 WT) were administered.

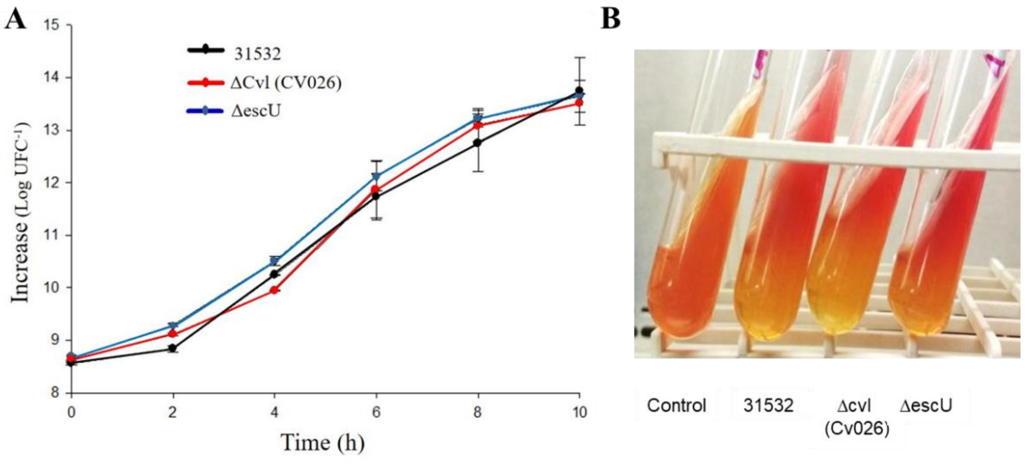

Figure S10. **A**: growth curve of the 313532 WT strain and its derived mutant strains. **B**: TSI medium for the degradation of sugars.

Table S1. Collection site and yield of dichloromethane extracts

Species name (Family)	Data collection sites	Yield (% w/w) *
Allium glandulosum Link & Otto (Amaryllidacae)-Brach	Estado de México, San Luis Huexotla. N 19° 28´ 02.55´´ W 98° 51´ 27.85´´	0.44
Allium glandulosum Link & Otto -Leave/Flower (Amaryllidacae)	Estado de México, San Luis Huexotla. N 19° 28´ 02.55´´ W 98° 51´ 27.85´´	1.29
Annona cherimola Mill. (Annonaceae)	Puebla, Puebla. 19°01'46''N., 98°10'12'' W. 2520 msnm	3.72
Argemone platyceras Link & Otto (Papaveraceae)	Estado de México, Texcoco. 13°32'52.1''N., 98°50'51'' W. 2268 msnm	2.99
Artemisa ludoviciana (Asteraceae)	Estado de México, Monte Tlaloc. 19°26'339''N., 98°46'29.6'' w. 2974 msnm	8.42
Buddleja parviflora H.B. & K (Scrophulariaceae)	Not available	1.72
Diphysa americana (Mill.) M. Sousa (Fabaceae)	Jaltocan, Hidalgo. N 21° 08´ 27´´ W 98° 32´ 03´´ 227 msnm	1.13
Euphorbia pulcherrima Willd. ex Klotzsch (Euphorbiacea)	Puebla, Atlixco. 18°53'34''N., 98°24'52'' w. 2129 msnm	3.15
Gnaphalium viscosum Kunth (Asteraceae)	Estado de México, Monte Tlaloc. 19°26'339''N., 98°46'29.6'' w. 2974 msnm	1.86
Gymnosperma glutinosum (Spreng.) Less (Asteraceae)	Estado de México, San Luis Huexotla. 19°28'31.3''N., 98°51'09.9'' w. 2336 msnm	11.61
Hibiscus sabdariffa L. (Malvaceae)	Guerrero, Mexico. 18°, 200´ 36.600´´ N; 99° 300´ 12.600´´ W.	0.93
Ipomoea dumosa (Benth.) L.O. Williams (Convolvulaceae)	Tepehuacán de Guerrero, Hidalgo. N 21° 03´ 22.8´´ W 98° 50´ 51.9´´1394 msnm	2.31
Jaltomata procumbens (Cav.) J.L. Gentry (Solanaceae)	Tepehuacán de Guerrero, Hidalgo. N 21° 00´ 45.3´´ W 98° 52´ 10.5´´1066 msnm	0.63
Loeselia mexicana (Lam.) Brand (Polemoniaceae)	Tequesquinahuac Texcoco. 19°27'58''N., 98°48'20.6'' w. 2593 m	2.99
Metastelma angustifolium Turcz (Apocynaceae)	Estado de México, San Luis Huexotla. N 19° 28´ 02.55´´ W 98° 51´ 27.85´´	2.99
Peperomia peltilimba C.DC. ex Trel. aff. *Peperomia aggravescens* Trel (Piperaceae)	Tlanchinol, Hidalgo. N 20° 57´ 05´´W 98° 39´ 47´´1000 msnm	4.58

Phaseolus coccineus L. (Fabaceae)	Tepehuacán de Guerrero, Hidalgo. N 2° 0´ 15.5´´ W 98° 51´ 49.1´´1365 msnm	1.50
Psidium guajava L. (Myrtaceae)	Not available	3.37
Rumex crispus L. (Polygonaceae)	Tepehuacán de Guerrero, Hidalgo. N 21° 03´ 25.1´´ W 98° 50´ 55´´1394 msnm	1.73
Saurauia scabrida Hemsl (Actinidiaceae)	Tepehuacán de Guerrero. N 21° 02´ 08.9´´ W 98° 51´ 24.7´´1259 msnm	3.07
Smilax regelii Killip & C.V. Morton (Smilacaceae)	Tlanchinol, Hidalgo. N 20° 57´ 05´´ W 98° 39´ 47´´1000 msnm	2.64
Tagetes lucida Cav. (Asteraceae)	General Manuel Ávila Camacho. 19°19'59.62''N., 98°48'12.7'' w. 2599 m	2.13
Tagetes micrantha Cav. (Asteraceae)	San Luis Huexotla, Texcoco. N 19° 28´ 02.55´´ W 98° 51´ 27.85´´	2.23
Xanthosoma sagittifolium (L.) Schott (Araceae)	Tepehuacán de Guerrero, Hidalgo. N 21° 01´ 15.5´´ W 98° 51´ 49.1´´ 1365 msnm	2.48

* Yield percentage = (weight of dichloromethane extract/weight of dry plant material) x 100

Table S2. Strains used in this study.

Strains	Features	Reference
PA14 WT	Reference strain isolated from a burn patient.	[82]
Δ*lasR*/Δ*rhlR*	PA14 mutant strain with Las and Rhl system disrupted.	[83]
Δ*pscC*	PA14 mutant strain with *pscC* gene of T3SS disrupted.	[82]
ATCC® 31532™	Reference strain	[78]
Δ*cviI* (CV026)	ATCC® 31532™ mutant strain with *cviI* gene of QS disrupted.	[78]
Δ*escU*	31532 mutant strain with *escU* gene of QS disrupted.	In this study

Table S3. Quantitative determination of flavonoids and terpenoids from anti-pathogenic extracts.

Extracts	Flavonoid (mg QE/g extract)	Terpenoids (mg UAE/g extract)
D. americana	102.43 ± 4.45 BC	550.23 ± 21.27C
H. sabdariffa	64.35 ± 0.74 A	296.56 ± 13.16A
J. procumbens	113.89 ± 7.04 C	335.05 ± 8.24A
P. coccineus	87.88 ± 5.19 B	536.9 ± 34.92 C
T. lucida	102.85 ± 9.46 BC	518.64 ± 16.69 C
L. mexicana	93.56 ± 10.39 BC	530.41 ± 6.41 C
P. peltilimba	185.0 ± 11.93 D	451.03 ± 3.42 B

Average ± SD. Different letter in each row means difference significative with One-way ANOVA and Tukey's comparative test; $\alpha \leq 0.05$. QE: Quercetin equivalents = (mg QE/mL of calibration curve/ extract concentration [0.25 mg/mL]) x 1000 x Dilution Factor. UAE: Ursolic acid equivalents = (mg UAE/mL of calibration curve/extract concentration [0.25 mg/mL]) x 1000 x Dilution Factor.

Table S4. Effect of plant extracts at subinhibitory concentration on the pathogenicity of *C. violaceum*

Treatments	Survival (%)	Abscess area (mm², mean ± S.E.)	Necrotic area (mm², mean ± S.E.)	Inoculation area (*log* CFU/g)
31532 wt	100	74.9 ± 18.0	31.7 ± 17.8	6.2 ± 0.4
ΔcviI (CVO26)	100	42.5 + 18.8	12.7 ± 5.2	6.4 ± 0.7
ΔescU	100	43.2 + 18.9	12.3 ± 6.6	6.0 ± 0.3
31532wt + plant extract [500 mg/mL]				
D. americana	100	39.3 ± 8.1	10.5 ± 1.4	5.7 ± 0.07
H. sabdariffa	100	25.1 ± 2.7	15.2 ± 4.1	5.6 ± 0.1
J. procumbens	100	55.8 ± 27.6	18.6 ± 9.3	5.8 ± 0.1
S. regelii	100	86.4 ± 36.9	4.2 ± 2.3	5.9 ± 0.2
P. coccineus	100	50.5 ± 7.5	18.3 ± 4.5	5.8 ± 0.2
T. lucida	100	37.4 ± 9.5	19.6 ± 4.3	6.1 ± 0.1
L. mexicana	100	25.4 ± 2.8	8.9 ± 3.4	6.0 ± 0.0
P. peltilimba	100	82.7 ± 4.6	25.7 ± 1.8	6.4 ± 0.1
R. crispus	100	51.8 ± 15.4	23.0 ± 10.8	6.1 ± 0.3
I. dumosa	100	59.7 ± 10.1	34.5 ± 3.5	6.5 ± 0.2
A. glandulosum- L/F	100	40.1 ± 10.2	8.1 ± 3.9	5.8 ± 0.1
A. ludoviciana	100	67.0 ± 20.3	17.5 ± 7.4	6.8 ± 0.03

The animals were inoculated subcutaneously with 10^9 CFU of the different strains. The abscess area was quantified at 24 h post-infection and the area of necrosis at 96 h, which corresponds to the maximum time of its formation ($n = 3$). The animals were sacrificed at 96 h, and the percentage of survival and CFU/g of tissues were determined. The test was carried out once with groups of three animals.
